# Spatial organization of FcγR and TLR2/1 on phagosome membranes differentially regulates their synergistic and inhibitory receptor crosstalk

**DOI:** 10.1038/s41598-021-92910-9

**Published:** 2021-06-28

**Authors:** Wenqian Li, Miao Li, Stephen M. Anthony, Yan Yu

**Affiliations:** 1 Department of Chemistry, Indiana University, Bloomington, IN 47405 USA; 2 Department of Molecular and Cellular Biochemistry, Indiana University, Bloomington, IN 47405 USA; 3 Department of Computational Biology and Biophysics, Sandia National Laboratories, Albuquerque, NM 87123 USA

**Keywords:** Monocytes and macrophages, Biophysics, Membrane biophysics, Nanoscale biophysics, Nanobiotechnology, Innate immune cells

## Abstract

Many innate immune receptors function collaboratively to detect and elicit immune responses to pathogens, but the physical mechanisms that govern the interaction and signaling crosstalk between the receptors are unclear. In this study, we report that the signaling crosstalk between Fc gamma receptor (FcγR) and Toll-like receptor (TLR)2/1 can be overall synergistic or inhibitory depending on the spatial proximity between the receptor pair on phagosome membranes. Using a geometric manipulation strategy, we physically altered the spatial distribution of FcγR and TLR2 on single phagosomes. We demonstrate that the signaling synergy between FcγR and TLR2/1 depends on the proximity of the receptors and decreases as spatial separation between them increases. However, the inhibitory effect from FcγRIIb on TLR2-dependent signaling is always present and independent of receptor proximity. The overall cell responses are an integration from these two mechanisms. This study presents quantitative evidence that the nanoscale proximity between FcγR and TLR2 functions as a key regulatory mechanism in their signaling crosstalk.

## Introduction

Innate immune cells use a variety of receptors to recognize invading pathogens and elicit appropriate immune responses. An increasing number of studies have shown that during host–pathogen interactions, two or more receptors often function simultaneously to orchestrate the overall immune responses^1–7^. This phenomenon is known as receptor crosstalk. This appears to be a key mechanism by which immune cells mount specific and selective responses to a diverse range of pathogens^8–11^. However, some microbial pathogens have evolved strategies that manipulate or disrupt this signaling crosstalk between receptors to evade host immune responses^12–16^. Extensive biochemical studies have identified the receptors and downstream signaling proteins involved in signaling crosstalk^17–20^. In contrast, the physical mechanisms that govern the interaction and signal integration between receptors are much less well known.

A general presumption is that receptors and downstream signaling proteins colocalize to enable signaling crosstalk through their physical interactions. For examples, Fc gamma receptor (FcγR) IIIa and interleukin-12 (IL-12) receptors have been shown to colocalize to enhance interferon (IFN)-γ production in natural killer cells^21^. IL-6Rα colocalizes and forms complexes with insulin-like growth factor (IGF)-I receptor on the plasma membrane of myeloma cells in their synergistic response to IL-6^22^. Dectin-1 and Toll-like receptor (TLR) 2/1 were also shown to colocalize during synergistic regulation of anti-fungal immune responses^23^. However, we have recently challenged this view by showing that nanoscale proximity, but not colocalization, between Dectin-1 and TLR 2/1 is required for their synergistic signaling crosstalk^24^. Despite discrepancies in the findings, all studies support the general notion that the spatial organization of innate immune receptors in membranes plays an important role in their signaling crosstalk.

In this study, we present new evidence supporting a vital role for spatial organization in FcγR and TLR2/1 crosstalk in macrophage cells. These two types of receptors recognize different microbial ligands. The TLR2/1 heterodimer recognizes lipopeptides on the cell walls of bacteria and yeast^25–28^, whereas FcγR recognizes immunoglobulin G (IgG) that is non-specifically adsorbed on pathogens^29^. Studies have shown that FcγRs can either enhance or inhibit TLR-mediated immune responses^30–34^. This dual role may derive from the fact that there are multiple different kinds of FcγRs. While all FcγRs recognize IgG, some of them are involved in immune cell activation and others in cell inhibition. FcγRIII and FcγRIV, for example, contain cytoplasmic immunoreceptor tyrosine-based activation motif (ITAM) and their ligation leads to the activation of immune cells^29,35^. In contrast, FcγRIIb contains immunoreceptor tyrosine-based inhibitory motif (ITIM) and its ligation leads to immune cell inhibition^29,35^. Some FcγRs, such as FcγRIIa, can be either activating or inhibitory depending on the ligand avidity^30–32^. The relationships involved can be quite complex. Some interactions can amplify the immune responses. Signaling from the activating receptor FcγRIII has been shown to amplify TLR4-induced cytokine production in peritoneal neutrophils^36^. FcγRs, including FcγRIIa, also synergize with TLR2 in triggering proinflammatory cytokine secretion in macrophages. This is known because the knockout or blockade of either type of receptors has been shown to cause a reduction in immune cell response^37–39^. TLR2 signaling in turn also enhances FcγR-mediated phagocytosis and cytokine secretion in monocytes^40^. Along with interactions that enhance the immune responses, there are others that reduce it. Inhibitory signals from low-avidity ligation of FcγRs down-regulate signaling of TLR2, TLR4, and TLR9^30–32^. For example, binding of FcγRIIa to anti-FcγRII antibody fragments inhibits TLR4-triggered cytokine responses in mouse macrophages^41^. Taken together, such studies clearly demonstrate the complex nature of the cross-regulation between FcγRs and TLRs in immune responses. However, the physical mechanisms governing the differential effects of FcγRs in receptor signaling crosstalk remain elusive.

In this study, we report that the synergistic and inhibitory signaling crosstalk between FcγR and TLR2/1 is differentially regulated by their spatial proximity on phagosome membranes. To show this, we fabricated synthetic particles as phagocytic targets on which ligands for FcγR and TLR2/1 were either homogeneously mixed or spatially separated by placement onto opposite sides of individual particles. We found that receptors in contact with such phagocytic targets are re-organized so that they are either in close proximity to one another or spatially separated, respectively. By using this geometric manipulation strategy, we revealed that FcγR can either enhance or inhibit TLR2-triggered inflammatory immune responses. This includes cytokine production and NF-κB activation, with a dependence on receptor proximity that differs drastically depending on the type of FcγR. The synergistic signaling between FcγR and TLR2/1 depends on receptor proximity and is diminished when the receptor pair is separated on the sub-micron scale. However, the inhibitory effect from FcγRIIb is always present and does not depend on the receptor proximity. As an overall result, immune cells can either be activated or inhibited depending on the spatial proximity between the receptors. Our results reveal a new physical mechanism underlying the complex signaling crosstalk between FcγR and TLR2/1.

## Results

### Fabrication and characterization of particles with different ligand presentations

**Figure 1:**
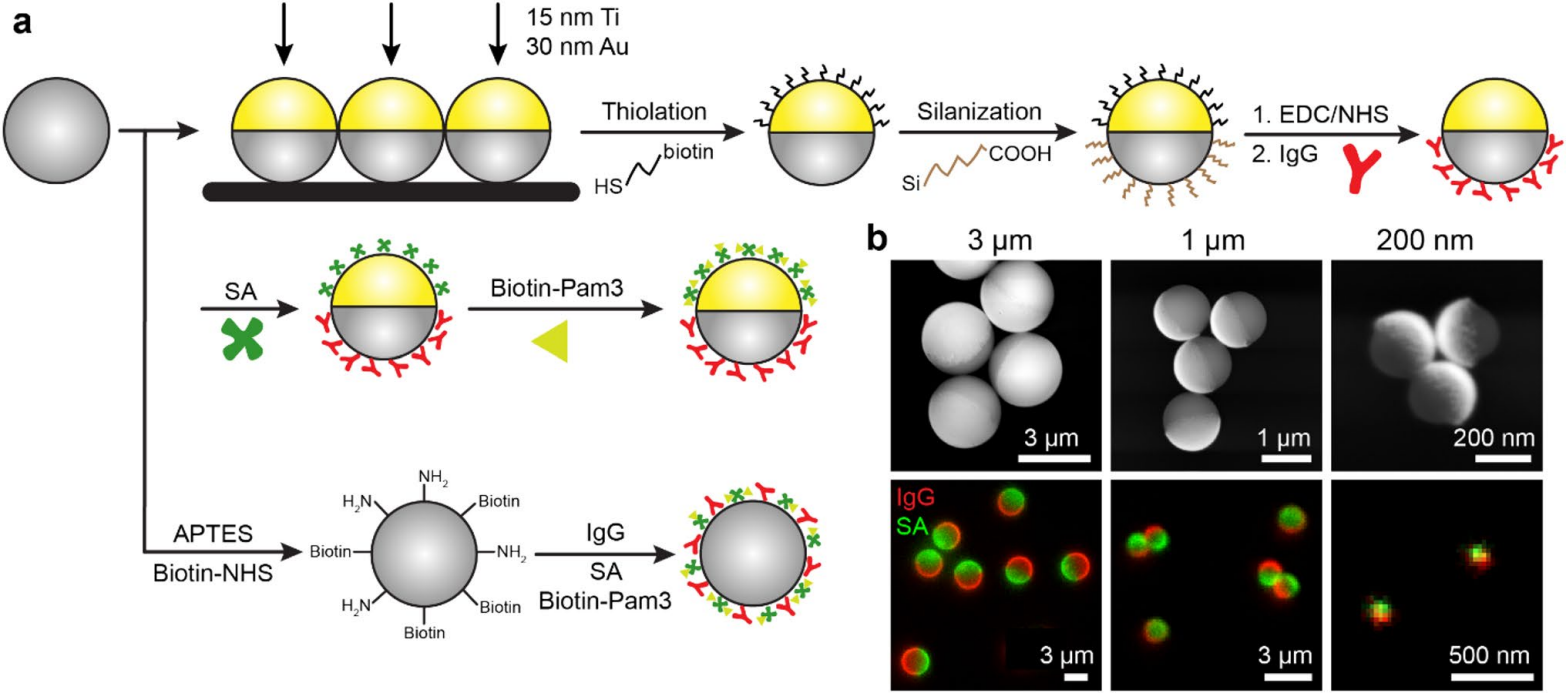
Fabrication and characterization of particles displaying different ligand arrangements. (**a**) Schematic illustration of fabrication procedures for Janus IgG/Pam3 (jIPam) and uniform IgG-Pam3 (uIPam) particles. Abbreviations used in the schematic: EDC: 1-ethyl-3-(-3-dimethylaminopropyl) carbodiimide hydrochloride; NHS: N-hydroxysuccinimide; SA: streptavidin; APTES: (3-Aminopropyl)triethoxysilane. (**b**) Scanning electron microscopy (top) and dual-color fluorescence (bottom) images showing jIPam particles of various sizes as indicated. In fluorescence images, IgG labeled with Alexa Fluor 647 and streptavidin (SA) labeled with Alexa Fluor 568 are shown in red and green, respectively.

In this study, we used RAW264.7 mouse macrophage cells, which express FcγRIII and FcγRIV, both of which activate immune responses, and inhibitory FcγRIIb^42^. IgG was used as ligand for FcγR and the synthetic triacylated lipopeptide Pam3CSK4 was ligand for TLR2/1^43,44^. Our first step was to create two types of particles with different spatial arrangements of ligands. On the first type of particle, IgG and Pam3CSK4 were spatially segregated onto opposite sides. This type is referred to as the Janus IgG/Pam3 (jIPam) particle (see Table 1 for the full list of particles and their abbreviation). It was designed to decouple FcγR and TLR2/1 on phagosome membranes. The jIPam particles (3 μm, 1 μm, and 200 nm in diameter) were prepared by first coating a thin gold film (30 nm thickness) onto one hemisphere of silica particles (schematic illustration in Fig. 1a). IgG was conjugated onto the silica hemisphere via carbodiimide crosslinking conjugation, and Pam3CSK4 on the gold-coated side via streptavidin–biotin linkage. The asymmetric coating of gold on the particles was confirmed using scanning electron microscopy (SEM) (Fig. 1b). By using fluorescently labeled IgG and streptavidin (as indicator for Pam3CSK4 conjugation), we confirmed that IgG and Pam3CSK4 were selectively functionalized on opposite sides of jIPam particles with no cross-contamination (Fig. 1b). On the second type of particles, IgG and Pam3CSK4 were homogeneously functionalized on silica particles (Fig. 1a). These uniform particles, referred to as uniform IgG-Pam3 particles (uIPam), are designed so that their ligands will induce mixing of FcγR and TLR2 on phagosome membranes. We tuned the conjugation reaction conditions so that the surface density of IgG and streptavidin (for anchoring Pam3CSK4), which was measured directly from their fluorescence emission intensity, is the same for both jIPam and uJPam particles. We matched the ligand density across different types of particles, so that we can clearly attribute differences in cell activation to the effect of ligand presentation on particles rather than to differences in ligand density.

**Table 1:** List of engineered particles and the abbreviations.

Abbreviation	Description of particle surface functionalization
jP	Bare Janus particles passivated with BSA and without ligand
jI	Janus particles with IgG on the silica side and BSA passivation on the gold-coated side
jPam	Janus particles with Pam3CSK4 on the gold-coated side and BSA passivation on the other
jIPam	Janus particles with IgG on the silica side and Pam3CSK4 on the gold-coated side
uIPam	Particles with uniformly coated IgG and Pam3CSK4

### Reorganization of pSyk and TLR2 on single phagosomes by ligand patterns on particles

**Figure 2:**
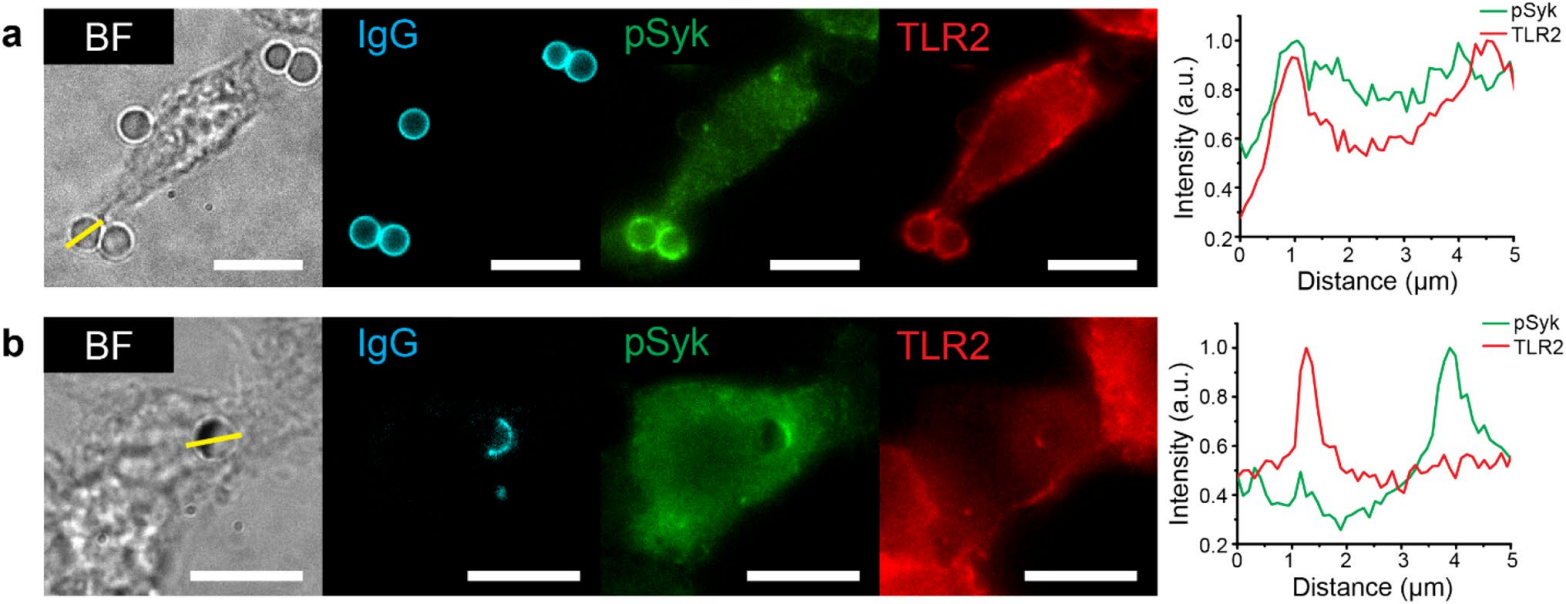
Spatial re-organization of pSyk and TLR2 on phagosome membranes. Representative bright-field (BF), fluorescence images, and line-scan plots showing IgG (cyan) on 3 μm particles and the recruitment of pSyk (green) and TLR2 (red) on phagosomes encapsulating uniform IgG-Pam3 (uIPam) particles (**a**) or Janus IgG/Pam3 (jIPam) particles (**b**) in fixed RAW264.7 macrophages. pSyk and TLR2 were immunostained with fluorescent primary antibodies. Images shown are representative of 90 phagosomes. All scale bars: 10 μm.

We next sought to determine whether FcγR and TLR2/1 are spatially rearranged on phagosome membranes by the two different ligand arrangements on the target particles. We stimulated RAW264.7 macrophages with jIPam and uIPam particles for 15 min and then immunostained TLR2 and phosphorylated spleen tyrosine kinase (pSyk) using fluorescently labeled antibodies. Syk is a cytoplasmic enzyme that is recruited to the phosphorylated immunoreceptor tyrosine-based activation motif (ITAM) of activated FcγRs, and then becomes phosphorylated for downstream signaling^45^. Thus, pSyk is a direct marker for activated FcγR. Immunofluorescence results show that both pSyk and TLR2 are homogenously distributed on phagosome membranes encapsulating uIPam particles (Fig. 2a). The intense accumulation of pSyk on phagosome membranes indicates that FcγRs are activated by IgG on the particles. On phagosomes encapsulating the jIPam particles, one can see clear segregation of pSyk and TLR2, with pSyk intensely concentrated on the IgG-conjugated side (appearing transparent under bright field) and TLR2 on the Pam3CSK4-conjugated side (appearing opaque under bright field due to the gold cap) (Fig. 2b). The TLR2 fluorescence emission appears dim in the images because it is partially blocked by the gold cap, but we confirmed that the segregation of pSyk and TLR2 is a representative phenomenon in over 90 phagosomes. These immunofluorescence results confirm our expectation that FcγR and TLR2 on phagosomes can be re-organized by the spatial arrangement of their ligands on phagocytosed particles.

### Cytokine secretion is modulated by the spatial organization of FcγRs and TLR2 on phagosomes

**Figure 3:**
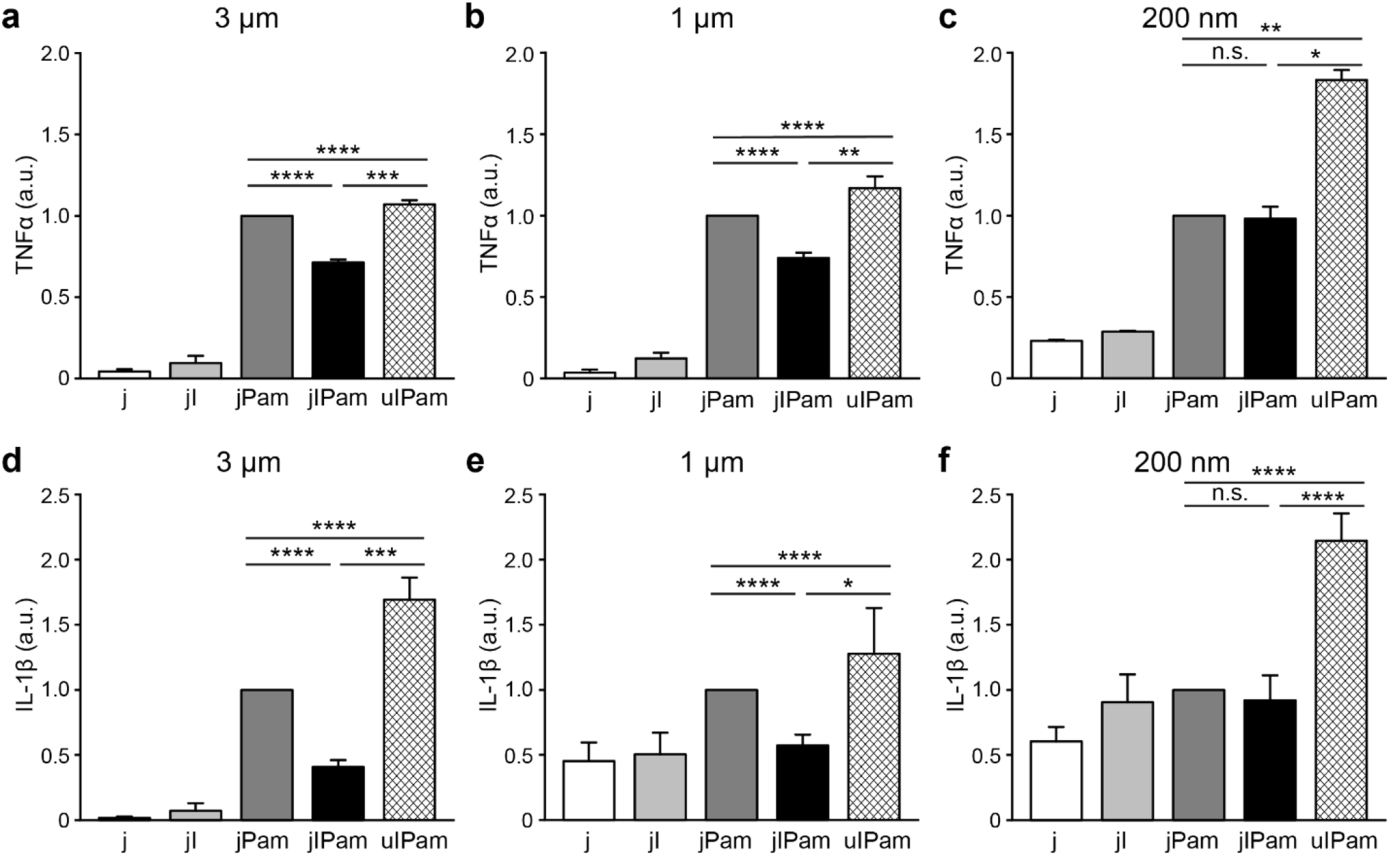
Measurements of cytokine secretion after particle stimulation. ELISA analysis of TNFα (**a–c**) and IL-1β (**d**–**f**) production in RAW264.7 macrophages after stimulation by different particles as indicated. Values were normalized against that from jPam particles in each sample group and are expressed as means ± standard errors. Data are representative of three independent experiments each performed in duplicate. Statistical significance is highlighted by p-values (Student’s t-test) as follows: ****P < 0.0001, ***P < 0.001, **P < 0.01, *P < 0.05, n.s. not significant.

Signaling crosstalk between FcγRs and TLRs has been shown to result in synergistic release of several inflammatory cytokines, including tumor necrosis factor (TNF) α, interleukin (IL)-1β, and IL-23^10,11,39^. Here, we measured the effect of receptor re-organization on the secretion of TNFα and IL-1β in RAW264.7 macrophages. In addition to jIPam and uIPam particles, we also tested several other types of control particles: (1) bared Janus particles passivated with bovine serum albumin (BSA) without any ligands (j), (2) Janus particles with IgG on one side and BSA passivation on the gold-coated side (jI), and (3) Janus particles with Pam3CSK4 on the gold-coated side and BSA passivation on the other (jPam) (see Table 1 for the full list of particles and their abbreviation). To stimulate RAW264.7 macrophage cells, we used the particle-to-cell ratio of 5:1 and 32:1 for 3 μm and 1 μm particles, respectively. The particle-to-cell ratio was increased for smaller particles to provide sufficient ligand stimulation. After cell stimulation by different particles, we measured the secretion of TNFα and IL-1β in supernatants using enzyme-linked immunosorbent assay (ELISA). Results from 3 μm and 1 μm particles share very similar trends (Fig. 3a,b,d,e). First, jPam particles induce significantly higher levels of TNFα and IL-1β than jI particles. This shows that TLR2/1 plays a more important role in stimulating cytokine secretion than FcγR under our experimental conditions. However, we did observe that the effect of TLR2/1 can be less dominant when its ligand density on particles was reduced. Second, the uIPam particles induce the maximal secretion of TNFα and IL-1β among all particle types, indicating that the synergistic activation of FcγR and TLR2 upregulates cytokine secretion in macrophages. This observation is consistent with a previous report that FcγRs and TLRs induce synergistic cytokine IL-1β release in dendritic cells (DCs)^10^. However, jIPam particles induced less secretion of TNFα and IL-1β than uIPam particles, indicating that when IgG and Pam3CSK4 are both present but spatially segregated on the jIPam particles, they do not induce synergistic signaling of FcγR and TLR2 (Fig. 3a). The drastically different cytokine responses in cells stimulated by uIPam particles versus by jIPam particles indicate that the crosstalk of FcγR and TLR2/1 depends on their spatial organization on phagosomes, which is, in turn, dictated by the way their ligands are presented on the particles. The close proximity between FcγR and TLR2/1 on phagosomes encapsulating uIPam particles leads to their synergistic up-regulation of the cytokine secretion. Surprisingly, the secretion of TNFα and IL-1β in cells stimulated by jIPam particles was even lower than those stimulated by jPam particles, suggesting that spatial segregation between the receptors causes a down-regulation of cytokine responses.

To investigate if the synergistic signaling between FcγR and TLR2/1 changes at nanoscale spatial separation, we performed the same TNFα and IL-1β ELISA measurements using 200 nm diameter particles for cell stimulation. A particle-to-cell ratio of 200:1 was used to provide sufficient ligand stimulation from those much smaller nanoparticles (Fig. 3c,f). Comparing the results from uIPam and jPam particles, it is clear that FcγR and TLR2/1 in close proximity have a synergistic crosstalk that up-regulates the cytokine secretion in RAW264.7 macrophage cells. This is consistent with our observations for larger 3 μm and 1 μm diameter particles. However, the jIPam particles induced similar levels of TNFα and IL-1β secretion as jPam particles. This is different from the observation of cytokine down-regulation induced by the 3 μm and 1 μm jIPam particles. Because the FcγR and TLR2/1 on phagosomes encapsulating the 200 nm jIPam particles are expected to be separated by 200 nm or less, this result suggests that a stronger synergistic signaling between FcγR and TLR2/1 as the spatial separation of receptors decreases.

In control experiments, we confirmed that the different types of particles are internalized by the macrophage cells at a similar efficiency after 16 h incubation, which is the same particle-cell incubation period used in TNFα and IL-1β measurements. This shows that the ligand arrangement on particles has negligible effect on the efficiency of long-time phagocytosis. It confirms that the different cytokine responses of cells are indeed a result of different ligand presentation on particles, rather than an effect of particle phagocytosis efficiency.

### NF-κB activation depends on the proximity of FcγR and TLR2 on phagosomes

**Figure 4:**
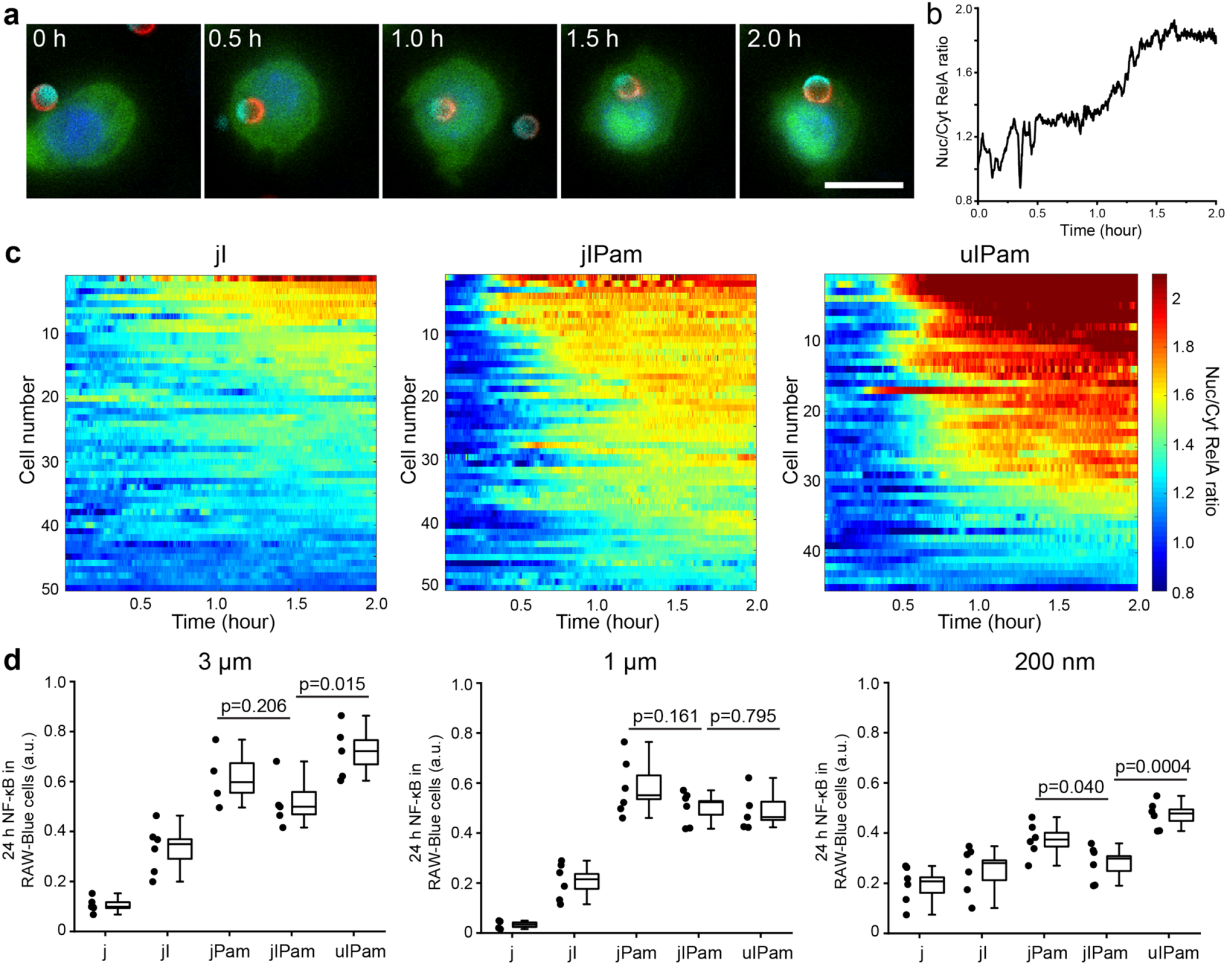
Measurements of NF-κB activation. (**a**) Time-lapsed images from live tracking of RelA translocation in RAW264.7 EGFP-RelA macrophages stimulated by 3 μm Janus IgG/Pam3 (jIPam) particles. Pseudo colors in fluorescence images: RelA (green), nucleus (blue), IgG (red), Pam3CSK4 (cyan). Scale bar: 10 μm. (**b**) Plot showing the Nuc/Cyt RelA ratio vs. time of the cell shown in (**a**). (**c**) Heatmaps showing RelA activation level in individual RAW264.7 EGFP-RelA macrophages as a function of time after stimulation by 3 μm jI particles, jIPam and uIPam particles. (**d**) Scatter plots showing the NF-κB activation levels in RAW-Blue cells stimulated by different types of particles as indicated for 24 h. Each box plot indicates the maximum to minimum, the median (horizontal line), and the standard deviation of the corresponding dataset. Data are representative of three independent experiments each performed in duplicate. Statistical significance is highlighted by p-values (Student’s t-test).

We next investigated the effect of FcγR and TLR2/1 crosstalk in the activation of nuclear factor κ-light-chain-enhancer of activated B cells (NF-κB). This is a protein complex found in the cytoplasm of resting cells that translocates into the nucleus of the cell after the immune cell is activated. There, it regulates DNA transcription^46^. To visualize the translocation of NF-κB in real time in living cells, we imaged the dynamics of GFP-RelA in RAW264.7 macrophages. RelA, also known as p65, is one of the five components in the NF-κB protein complex^47^. We also stained the nucleus of cells with Hoechst dye in order to distinguish the distribution of RelA in the nucleus from that in the cell cytoplasm in live cell fluorescence imaging. Using 3 μm jIPam particles as an example, we observed that translocation of GFP-RelA became obvious about 1 h after the initial particle-cell contact when FcγR and TLR2/1 were activated (SI Video 1 and Fig. 4a). To quantify the RelA translocation, we calculated the ratio of RelA fluorescence intensity in the cell nucleus to that in the cytoplasm (referred to as “Nuc/Cyt RelA ratio”) as a function of time (Fig. 4b). From the single cell plots, we observed that the Nuc/Cyt RelA ratio gradually increased after cell internalization of particles, indicating NF-κB activation of cells (Fig. 4b). In most cells, the RelA level inside cell nuclei reached a plateau about 1–1.5 h after initial stimulation. This observation of dynamic RelA activation is consistent with the previous report that the nuclear translocation of RelA reaches a maximum at 1 h and then decreases after 5 h^48,49^. Such dynamics of NF-κB activation in macrophage cells is further demonstrated by the heatmap, in which the Nuc/Cyt RelA ratios of single cells (N > 50) are plotted against time, and color-coded (Fig. 4c). It is clear that the RelA activation varies among cells, reflecting the intrinsic cell-to-cell heterogeneity. The uIPam particles overall triggered a significantly higher level of NF-κB activation than jIPam particles, and both uIPam and jIPam particles induced more NF-κB activation than jI particles. This result demonstrates that the FcγR and TLR2 synergistically regulate NF-κB activation when both receptors are brought into proximity, but their signaling synergy is disrupted when both receptors are spatially separated.

Based on those live cell observations of the dynamics of RelA, we then measured the NF-κB activation levels after particle stimulation in RAW-Blue reporter cells for 24 h. This allows us to evaluate the overall NF-κB activation from a large population of cells. In this measurement, NF-κB activation in RAW-Blue cells leads to secretion of embryonic alkaline phosphatase (SEAP)^50^, which we detected in supernatants using a QUANTI-Blue colorimetric enzyme assay. We first found that jIPam particles stimulated a lower level of NF-κB activation than jPam particles (Fig. 4d). This again indicates that the spatial segregation of IgG and Pam3 on the Janus particles lead to down regulation of FcγR and TLR2/1 synergy. This phenomenon is consistent with our observation in the NF-κB measurements. The 3 μm and 200 nm uIPam particles induced the highest level of NF-κB activation, indicating the synergy between FcγR and TLR2/1. However, we repeatedly found that NF-κB activation by 1 μm uIPam particles is similar to that by jIPam particles (Fig. 4d). The cause of this is unclear.

### The inhibitory and stimulatory effect of FcγR and TLR2/1 synergy

**Figure 5:**
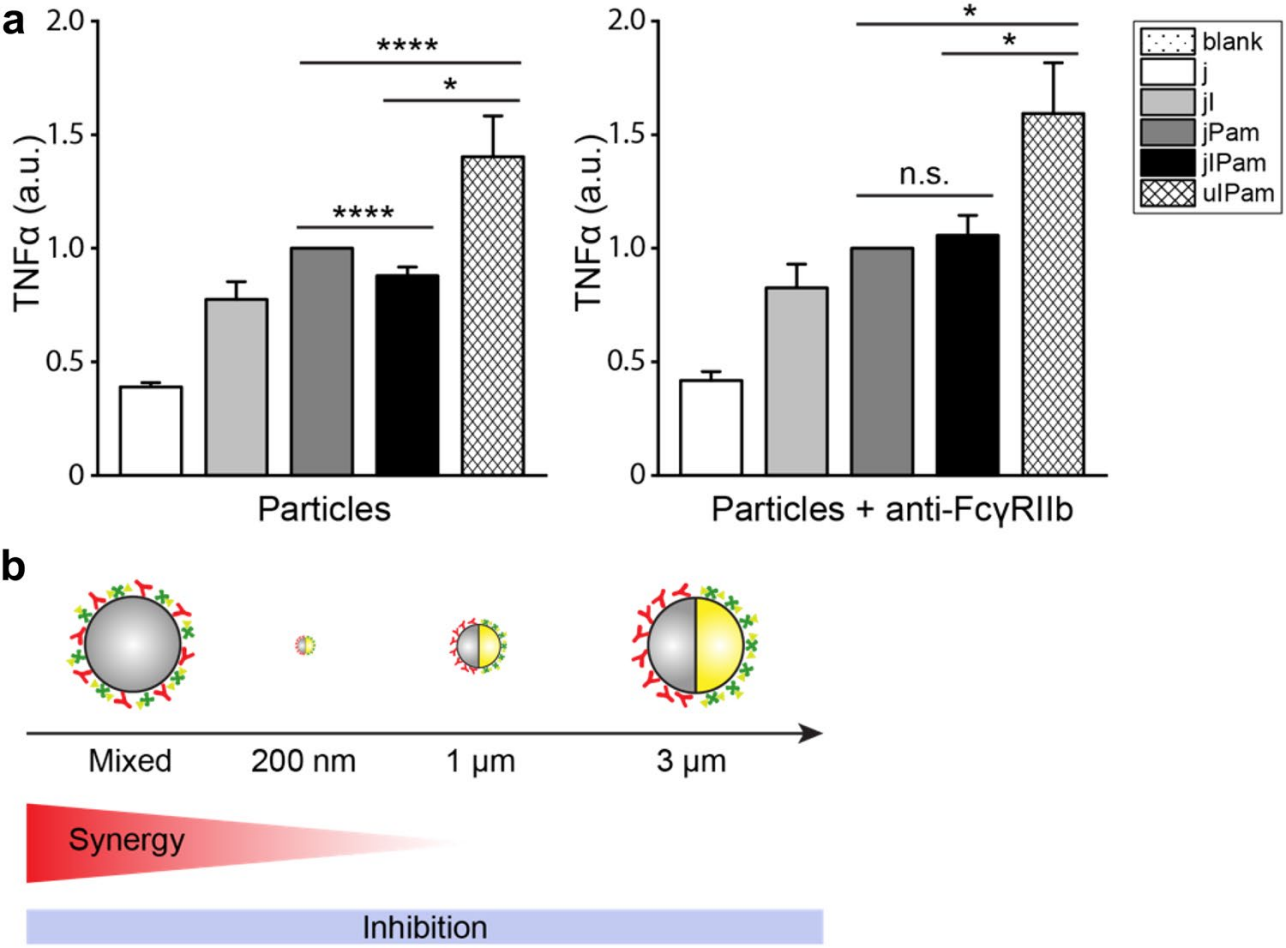
Effect of FcγRIIb blockade on TNFα secretion and proposed mechanism. (**a**) ELISA analysis of TNFα production in RAW264.7 macrophages that were blocked by anti-FcγRIIb antibody K9.361 and then stimulated by 3 µm particles with various ligand presentations as indicated. Results were normalized against that from jPam particles and are expressed as means ± standard errors. Data are representative of three independent experiments each performed in duplicate. Statistical significance is highlighted by p-values (Student’s t test) as follows: ****P < 0.0001, *P < 0.05, n.s. not significant. The p-value for uIPam particle samples with and without anti-FcγRIIb is 0.140. (**b**) Schematic illustration showing the proposed mechanism by which the physical segregation between IgG and Pam3CSK4 differentially affects the synergistic and inhibitory crosstalk between FcγR and TLR2/1.

We next sought to understand why uIPam particles induce synergistic signaling between FcγR and TLR2/1, but jIPam particles appear to down regulate it. RAW264.7 macrophage cells express FcγRIII and FcγRIV, both activating receptors, and FcγRIIb. All three types of receptors recognize IgG^42^. It was shown previously that blocking all FcγRII reduces IL-1β release in dendritic cells, but blocking of only FcγRIIb increases IL-1β secretion. This observation suggests that the signaling crosstalk between FcγRs and TLRs in dendritic cells is an integrated effect caused by both activating FcγRs and inhibitory FcγRIIb^10^. We asked if the same synergistic mechanism is also involved in our observation of the up- and down-regulation of RAW264.7 macrophage activation by the particles. To answer this question, we blocked the inhibitory FcγRIIb in cells with specific antibody K9.361^51,52^ and measured the TNFα secretion upon stimulation by 3 μm particles. We made three important observations. First, the TNFα secretion induced by uIPam particles increased from 1.402 ± 0.441 (ave ± s.d.) and 1.923 ± 0.660 after FcγRIIb blockade (Fig. 5a), which confirms that FcγRIIb indeed exerts an inhibitory effect on the synergistic release of TNFα. Second, with FcγRIIb blockade, the TNFα secretion induced by jIPam particles also increased significantly to a similar level as that by jPam particles. This is different from the case of no FcγRIIb blockade when jIPam particles induced a lower level of TNFα than jPam particles. This shows that while the positive synergistic effect of FcγR on TLR2-stimulated TNFα secretion disappeared when their ligands are spatially separated on the 3 μm jIPam particles, the inhibitory effect of FcγRIIb is present regardless of the spatial distribution of the ligands. In addition, uIPam particles induced the highest level of TNFα release regardless of FcγRIIb blockade, indicating that the positive synergistic signaling from FcγR and TLR2/1 is independent of FcγRIIb.

Based on our findings, we propose a model to explain quantitatively how the signaling crosstalk between FcγR and TLR2/1 depends on the spatial proximity of these two kinds of receptors (schematic illustration in Fig. 5b). The synergistic positive signaling between the receptor pair decreases as their proximity increases, but the inhibitory effect from FcγRIIb is independent of receptor proximity (Fig. 5b). Therefore, when FcγR and TLR2/1 are uniformly mixed, their synergistic positive signaling dominates the inhibitory effect, leading to overall enhanced cytokine responses than TLR2/1 activation alone. When FcγR and TLR2/1 are separated on a phagosome encapsulating 200 nm jIPam particles, the proximity between receptors is increased to the range of 0–200 nm. The overall synergistic positive signaling decreases to the level that it counterbalances the inhibitory effect, leading to the observation of similar overall signaling induced by jIPam and jPam particles. As the spatial separation between receptors further increases on phagosomes that encapsulate 1 μm and 3 μm jIPam particles, synergistic positive signaling between the receptors further decreases, so the inhibitory effect dominates. As a result, cytokine responses induced by 1 μm and 3 μm jIPam particles are less than that by jPam particles.

## Discussion

In this study, we investigated how the spatial organization of FcγR and TLR2/1 on phagosomes regulates their signaling crosstalk by using a geometric manipulation strategy that we developed previously^24^. We engineered particles as phagocytic targets on which the IgG and Pam3CSK4 ligands are either homogeneously mixed over the surface of the particle or spatially segregated onto the surfaces of its opposite hemispheres. We showed that the FcγRs and TLR2/1 receptors on phagosomes encapsulating such engineered particles are spatially re-organized to follow the ligand patterns on the surfaces of the particles. We studied the effects of this geometric manipulation using quantitative measurements of cytokine secretion and NF-κB activation in RAW264.7 macrophage cells. We obtained two important results. First, FcγRs and TLR2/1 receptors have synergistic signaling crosstalk when both receptors are within nanoscale proximity, resulting in enhanced secretion of TNFα and IL-1β as well as NF-κB activation. However, this synergistic signaling decreases with increased receptor separation. Second, the inhibitory receptor FcγRIIb negatively regulates TLR2/1-triggered immune responses. Unlike the synergistic effects produced by other forms of FcγRs, the inhibitory effect of FcγRIIb occurs even if this receptor is spatially segregated from TLR2/1. Our results reveal that the synergistic and inhibitory signaling crosstalk between FcγRs and TLR2/1 have distinctly different dependences on their spatial organization on phagosomes. Some previous studies had already suggested that FcγRs and TLRs function synergistically in innate immune regulation^11,37,39^. FcγRs are also known to contain both stimulatory and inhibitory subtypes^29,35^. Unlike previous studies, our findings here have identified a new physical mechanism underlying the complex signaling crosstalk between this receptor pair.

The key conclusion from this study is that the nanoscale proximity between FcγR and TLR2 functions as an important regulatory mechanism in their signaling crosstalk. This reinforces our earlier finding that Dectin-1 and TLR2/1 must reside in nanoscale proximity to synergistically regulate the innate immune responses of macrophage cells^24^. Interestingly, Dectin-1 and FcγR both contain ITAM domains in their cytoplasmic tails and share several associated adaptor proteins, such as spleen tyrosine kinase (Syk), in their signaling pathways. Both studies support the general theory that the physical proximity between ITAM-dependent signaling units and myeloid differentiation primary response (MyD)88-dependent TLR signaling units regulates their functional crosstalk and the subsequent innate immune responses.

The spatial proximity mechanism we have discovered provides insights of general importance for understanding host cell-pathogen interactions. It particularly sheds light on the question of how the distinctive physiochemical properties and spatial distributions of microbial ligands might modulate the responses of the host cell. Prior studies have shown that the crosstalk between innate immune receptors is modulated by the type, molecular structure, and geometry of microbial ligands. For example, diacylated lipoproteins induce dimerization and cooperative signaling of TLR2 with TLR6, but triacylated lipoproteins instead activate TLR2 with TLR1^53^. Lipopolysaccharide (LPS) molecules that contain different shapes of the lipid A component were also shown to activate different TLR heterodimers and subsequently different responses in mononuclear cells^54,55^. We have demonstrated here, and in our previous study, that the nanoscale proximity between receptors functions as another key regulatory mechanism for innate receptor crosstalk^24,56^. In addition to revealing a new role for spatial proximity in receptor crosstalk, our studies have also demonstrated that the geometric manipulation strategy we have established is more generally applicable for investigating the relationship between receptor crosstalk and the spatial organization of receptors on phagosome membranes in living cells.

## Methods

### Reagents and cells

3 μm and 1 μm silica particles were from Spherotech (Lake Forest, IL). The estimated average diameter of the particles was 3.15 μm and 1.24 μm, respectively. 200 nm silica particles were from nanoComposix (San Diego, CA). HS-PEG-biotin (3400 Da) and silane-PEG-COOH (5000 Da) were from Nanocs (New York, NY). Sodium borohydride (NaBH_4_), sodium orthovanadate (Na_3_VO_4_), 2-(N-morpholino)ethanesulfonic acid (MES), 4-(2-hydroxyethyl)-1-piperazineethanesulfonic acid (HEPES), ( +)-biotin N-hydroxysuccinimide ester (biotin-NHS) and IgG from rabbit serum were from Sigma-Aldrich (St. Louis, MO). 1-(3-Dimethylaminopropyl)-3-ethylcarbodiimide hydrochloride (EDC) and 3-aminopropyltriethoxysilane (APTES) were from Acros Organics (Fair Lawn, NJ). Pam3CSK4-biotin, Normocin, Zeocin and QUANTI-Blue were from InvivoGen (San Diego, CA). Paraformaldehyde (PFA) was from Avantor (Radnor, PA). Alexa Fluor 488 conjugated phospho-Syk (Tyr525/526) rabbit monoclonal antibody (4349S) was from Cell Signaling Technology (Danvers, MA). Alexa Fluor succinimidyl esters, Hoechst, streptavidin (SA), eFluor 660 conjugated TLR2 monoclonal antibody (50–9021-82), Alexa Fluor 405 conjugated goat-anti-rabbit IgG secondary antibody, tumor necrosis factor α (TNFα) mouse uncoated ELISA kit with plates (88–7324-86) and interleukin (IL)-1β mouse uncoated ELISA kit (88–7013-88) were purchased from Invitrogen (Carlsbad, CA). Sulfo-N-hydroxysulfosuccinimide (sulfo-NHS), trypan blue and Pierc Fab micro preparation kit were purchased from Thermo Fisher Scientific (Waltham, MA). 24-well glass bottom plates (P24G-1.5–10-F) were from MatTek Corporation (Ashland, MA); 96-well glass bottom plates (P96-1.5H-N) were from Cellvis (Mountain View, CA). RAW264.7 macrophages were purchased from ATCC (Manassas, VA) and cultured in Dulbecco’s modified Eagle’s medium (DMEM) containing with 10% fetal bovine serum (FBS), 2 mM l-glutamine, 100 U/mL penicillin and 100 μg/mL streptomycin at 37 °C and 5% CO_2_. Anti-mouse FcγRIIb monoclonal antibody K9.361 was provided by Prof. Fred D. Finkelman at University of Cincinnati Medical Center. RAW264.7 EGFP-RelA cells were provided by Dr. Iain D. C. Fraser at National Institutes of Health and cultured in DMEM containing with 10% FBS, 2 mM l-glutamine and 10 mM HEPES at 37 °C and 5% CO_2_. RAW-Blue cells were purchased from InvivoGen (San Diego, CA) and cultured in Dulbecco’s modified Eagle’s medium (DMEM) containing with 10% heat-inactivated FBS, 2 mM l-glutamine, 100 U/mL penicillin, 100 μg/mL streptomycin, 100 μg/mL Normocin and 200 μg/mL Zeocin at 37 °C and 5% CO_2_.

### Particle fabrication and characterization

To fabricate bifunctional Janus IgG-Pam3CSK4 particles, silica particles were cleaned with piranha solution for 15 min and prepared into monolayers on pre-cleaned glass microscope slides. Particle monolayers were coated sequentially with layers of titanium (15 nm) and gold (30 nm) using Temescal Electron beam Evaporator 4 (Materials Research Laboratory, University of Illinois at Urbana-Champaign). Particles were sonicated off glass slides, cleaned with 0.6 NaBH_4_ for 1 h and then incubated with 1 mM HS-PEG-biotin in ethanol overnight. Particles were next functionalized with 0.4 mM silane-PEG-COOH in 95% ethanol solution for 3.5 h and dried for 30 min. Particles were activated with 10 mM EDC and 25 mM sulfo-NHS in 0.1 M MES buffer (pH 6.0) for 25 min and then conjugated with 16 μg/mL IgG in phosphate buffered saline (PBS) buffer for 2 h. Particles were incubated with 2 μg/mL SA in PBS buffer for 30 min and then incubated with 100 ng/mL Pam3CSK4-biotin in PBS buffer for 1 h.

To fabricate bifunctional uniform IgG-Pam3CSK4 particles, silica particles were first functionalized by 2% APTES in ethanol overnight and then partially modified by 1 mM biotin-NHS in 0.1 M NaHCO_3_ solution for 1 h. Particles were next activated by 2.5% glutaraldehyde in PBS buffer for 1 h and then conjugated with IgG in PBS buffer for 1 h. Particles were finally incubated with SA in PBS buffer for 30 min and then Pam3CSK4-biotin in PBS buffer for 1 h.

The concentrations of 3 μm and 1 μm particles were estimated using hemocytometer. The concentration of 200 nm particles was measured using ZetaView Nanoparticle Tracking Analyzer. Morphology of gold-coated Janus particles was characterized using scanning electron microscopy. Both IgG and SA were fluorescently labeled by different Alexa Fluor dyes and thus the surface density of ligands on different particles can be matched based on fluorescence intensity. Spatial distribution and fluorescence intensity of ligands were characterized using a Nikon Eclipse Ti microscope equipped with an Andor iXon3 EMCCD camera and a Nikon Apo 100 × /1.49 TIRF objective.

### Measurement of phagocytosis efficiency

RAW264.7 macrophages were plated at 3 × 10^6^ cells per well in a glass bottom 24-well plate in complete DMEM media for 5 h and serum starved for 3 h. Particles were added to cells and incubated for stimulation for 16 h at a particle: cell ratio of 5:1, 32:1, 200:1 for 3 μm, 1 μm and 200 nm, respectively. The supernatants were collected for cytokine measurements. The remaining samples containing cells and particles were used for phagocytosis efficiency measurements. To count phagocytosis efficiency of IgG-coated particles, 1 μg/mL Alexa Fluor 405 conjugated goat-anti-rabbit IgG antibody was added to the samples to label particles outside of cells. The total number of extracellular particles was counted in epi-fluorescence images. The number of internalized particles was calculated by subtracting the number of extracellular particles from the total number of particles added in the sample, and phagocytosis efficiency was obtained by dividing the number of internalized particles by the total number of particles. To count phagocytosis efficiency of particles without IgG coating, trypan blue was added to the samples at a final concentration of 0.0015% and incubated at room temperature for 1 h to quench the fluorescence of Alexa Fluor 568 labeled streptavidin on particles. The internalized particles remained fluorescent, and their total number was counted in epi-fluorescence images. Phagocytosis efficiency was then calculated by dividing the number of internalized particles by the total number of particles added in each sample.

### Immunofluorescence imaging

RAW264.7 macrophages were seeded on 30 mm glass coverslips overnight and then serum starved for 3 h. Cells were stimulated with particles at 37 °C for 15 min. Samples were sequentially fixed with 2% PFA on ice for 5 min, permeabilized with acetone at −20 °C for 5 min, blocked with 2% BSA at room temperature for 30 min and stained with 2 μg/mL Alexa Fluor 488 conjugated phospho-Syk antibody and 2 μg/mL eFluor 660 conjugated TLR2 antibody at room temperature for 1 h. Fluorescence images were acquired using Nikon Eclipse Ti microscope equipped with an Andor iXon3 EMCCD camera and a Nikon Apo 100 × /1.49 TIRF objective.

### Measurements of cytokine secretion

Cell supernatants were collected during the measurement of phagocytosis efficiency as described above. TNFα and IL-1β level in the supernatants was measured using ELISA kits according to manufacturer instructions. Absorbance at 450 nm and 750 nm was measured using BioTek Synergy H1 microplate reader.

For FcγRIIb blockade experiments, anti-FcγRIIb antibody K9.361 was digested and purified into Fab fragments by Pierc Fab micro preparation kit according to manufacturer instructions. Cells were incubated with 100 μg/mL the Fab fragments of anti-FcγRIIb antibody on ice for 30 min before particle stimulation.

### Measurements of NF-κB activation

In Rel-A nuclear translocation assay, RAW264.7 EGFP-RelA cells were plated at 4 × 10^5^ cells per well on a 96-well glass bottom plate overnight and then serum starved for 3 h. Cells were stimulated with the same amounts of different 3 μm particles at 5:1 particle: cell ratio, and incubated at 37 °C for 0.5, 1.0, 1.5 and 2.0 h. Cells were fixed by 2% PFA on ice for 5 min. Nuclei of cells were then counterstained with 2 μg/mL Hoechst at room temperature for 15 min. Fluorescence images were acquired using a Nikon Eclipse Ti microscope equipped with an Andor iXon3 EMCCD camera and a Nikon Plan Apo 40 × /0.95 objective. Twenty images were taken at different locations of each sample. Images were analyzed using ImageJ following a previously reported method^24,57^.

QUANTI-Blue colorimetric enzyme assay: RAW-Blue cells were added at 1 × 10^5^ cells per well in a 96-well plate and sequentially the same amounts of different particles at a particle: cell ratio of 5:1, 32:1, 200:1 for 3 μm, 1 μm and 200 nm particles, respectively, were added into each well. Cells and particles were incubated at 37°C for 24 h and supernatants were collected. 20 μL cell supernatant and 180 μL QUANTI-Blue solution were added into a 96-well plate and incubated at 37 °C for 3 h. Absorbance at 655 nm was measured by BioTek Synergy H1 microplate reader.

## Supplementary Information

Supplementary Video 1.

Supplementary Information 1.

## References

[1] Gantner BN, Simmons RM, Canavera SJ, Akira S, Underhill DM (2003). Collaborative induction of inflammatory responses by Dectin-1 and Toll-like receptor 2. J. Exp. Med..

[2] van Egmond M, Vidarsson G, Bakema JE (2015). Cross-talk between pathogen recognizing Toll-like receptors and immunoglobulin Fc receptors in immunity. Immunol. Rev..

[3] Kawai T, Akira S (2010). The role of pattern-recognition receptors in innate immunity: update on Toll-like receptors. Nat. Immunol..

[4] Kawai T, Akira S (2011). Toll-like receptors and their crosstalk with other innate receptors in infection and immunity. Immunity.

[5] Trinchieri G, Sher A (2007). Cooperation of Toll-like receptor signals in innate immune defence. Nat. Rev. Immunol..

[6] Thaiss CA, Levy M, Itav S, Elinav E (2016). Integration of innate immune signaling. Trends Immunol..

[7] Underhill DM (2007). Collaboration between the innate immune receptors dectin-1, TLRs, and Nods. Immunol. Rev..

[8] Nakamura K (2008). Toll-like receptor 2 (TLR2) and dectin-1 contribute to the production of IL-12p40 by bone marrow-derived dendritic cells infected with *Penicillium marneffei*. Microbes Infect..

[9] Netea MG, Brown GD, Kullberg BJ, Gow NA (2008). An integrated model of the recognition of *Candida albicans* by the innate immune system. Nat. Rev. Microbiol..

[10] Bakema JE (2015). Antibody-opsonized bacteria evoke an inflammatory dendritic cell phenotype and polyfunctional Th cells by cross-talk between TLRs and FcRs. J. Immunol..

[11] den Dunnen J (2012). IgG opsonization of bacteria promotes Th17 responses via synergy between TLRs and FcγRIIa in human dendritic cells. Blood.

[12] Hajishengallis G, Lambris JD (2011). Microbial manipulation of receptor crosstalk in innate immunity. Nat. Rev. Immunol..

[13] Lambris JD, Ricklin D, Geisbrecht BV (2008). Complement evasion by human pathogens. Nat. Rev. Microbiol..

[14] Finlay BB, McFadden G (2006). Anti-immunology: evasion of the host immune system by bacterial and viral pathogens. Cell.

[15] Bowie AG, Unterholzner L (2008). Viral evasion and subversion of pattern-recognition receptor signalling. Nat. Rev. Immunol..

[16] da Silva FP (2007). CD16 promotes *Escherichia coli* sepsis through an FcRγ inhibitory pathway that prevents phagocytosis and facilitates inflammation. Nat. Med..

[17] Marco C (2007). All roads lead to CARD9. Nat. Immunol..

[18] Inoue M, Shinohara ML (2014). Clustering of pattern recognition receptors for fungal detection. PLoS Pathog..

[19] Cabodi S (2009). Convergence of integrins and EGF receptor signaling via PI3K/Akt/FoxO pathway in early gene Egr-1 expression. J. Cell. Physiol..

[20] Wang CC, Cirit M, Haugh JM (2009). PI3K-dependent cross-talk interactions converge with Ras as quantifiable inputs integrated by Erk. Mol. Syst. Biol..

[21] Kondadasula SV (2008). Colocalization of the IL-12 receptor and FcγRIIIa to natural killer cell lipid rafts leads to activation of ERK and enhanced production of interferon-γ. Blood.

[22] Abroun S (2004). Receptor synergy of interleukin-6 (IL-6) and insulin-like growth factor-I in myeloma cells that highly express IL-6 receptor α. Blood.

[23] Shin DM (2008). *Mycobacterium abscessus* activates the macrophage innate immune response via a physical and functional interaction between TLR2 and dectin-1. Cell. Microbiol..

[24] Li W, Yan J, Yu Y (2019). Geometrical reorganization of Dectin-1 and TLR2 on single phagosomes alters their synergistic immune signaling. Proc. Natl. Acad. Sci. USA.

[25] Aliprantis AO (1999). Cell activation and apoptosis by bacterial lipoproteins through Toll-like receptor-2. Science.

[26] Takeuchi O (1999). Differential roles of TLR2 and TLR4 in recognition of gram-negative and gram-positive bacterial cell wall components. Immunity.

[27] Underhill DM (1999). The Toll-like receptor 2 is recruited to macrophage phagosomes and discriminates between pathogens. Nature.

[28] Cuevas CD, Ross SR (2014). Toll-like receptor 2-mediated innate immune responses against Junin virus in mice lead to antiviral adaptive immune responses during systemic infection and do not affect viral replication in the brain. J. Virol..

[29] Nimmerjahn F, Ravetch JV (2008). Fcγ receptors as regulators of immune responses. Nat. Rev. Immunol..

[30] Hamerman JA, Lanier LL (2006). Inhibition of immune responses by ITAM-bearing receptors. Sci. STKE.

[31] Ivashkiv LB (2008). A signal-switch hypothesis for cross-regulation of cytokine and TLR signalling pathways. Nat. Rev. Immunol..

[32] Hirsch I, Janovec V, Stranska R, Bendriss-Vermare N (2017). Cross talk between inhibitory immunoreceptor tyrosine-based activation motif-signaling and Toll-like receptor pathways in macrophages and dendritic cells. Front. Immunol..

[33] Blank U, Launay P, Benhamou M, Monteiro RC (2009). Inhibitory ITAMs as novel regulators of immunity. Immunol. Rev..

[34] Underhill DM, Goodridge HS (2007). The many faces of ITAMs. Trends Immunol..

[35] Guilliams M, Bruhns P, Saeys Y, Hammad H, Lambrecht BN (2014). The function of Fcγ receptors in dendritic cells and macrophages. Nat. Rev. Immunol..

[36] Rittirsch D (2009). Cross-talk between TLR4 and FcγReceptorIII (CD16) pathways. PLoS Pathog..

[37] Vogelpoel LT (2015). FcγRIIa cross-talk with TLRs, IL-1R, and IFNγR selectively modulates cytokine production in human myeloid cells. Immunobiology.

[38] Hunt D, Drake LA, Drake JR (2018). Murine macrophage TLR2-FcγR synergy via FcγR licensing of IL-6 cytokine mRNA ribosome binding and translation. PLoS ONE.

[39] Vogelpoel LT (2014). Fc gamma receptor-TLR cross-talk elicits pro-inflammatory cytokine production by human M2 macrophages. Nat. Commun..

[40] Shah P (2013). Toll-like receptor 2 ligands regulate monocyte Fcγ receptor expression and function. J. Biol. Chem..

[41] Ben MS (2014). Shifting FcγRIIA-ITAM from activation to inhibitory configuration ameliorates arthritis. J. Clin. Invest..

[42] Bruhns P (2012). Properties of mouse and human IgG receptors and their contribution to disease models. Blood.

[43] Jin MS (2007). Crystal structure of the TLR1-TLR2 heterodimer induced by binding of a tri-acylated lipopeptide. Cell.

[44] Schromm AB (2007). Physicochemical and biological analysis of synthetic bacterial lipopeptides: validity of the concept of endotoxic conformation. J. Biol. Chem..

[45] Mocsai A, Ruland J, Tybulewicz VL (2010). The SYK tyrosine kinase: a crucial player in diverse biological functions. Nat. Rev. Immunol..

[46] Kingeter LM, Lin X (2012). C-type lectin receptor-induced NF-κB activation in innate immune and inflammatory responses. Cell. Mol. Immunol..

[47] Oeckinghaus A, Ghosh S (2009). The NF-κB family of transcription factors and its regulation. Cold Spring Harb. Perspect. Biol..

[48] Sung M-H (2014). Switching of the relative dominance between feedback mechanisms in lipopolysaccharide-induced NF-κB signaling. Sci. Signal..

[49] Drechsler Y, Chavan S, Catalano D, Mandrekar P, Szabo G (2002). FcγR cross-linking mediates NF-κB activation, reduced antigen presentation capacity, and decreased IL-12 production in monocytes without modulation of myeloid dendritic cell development. J. Leukoc. Biol..

[50] Lewis RE, Liao G, Young K, Douglas C, Kontoyiannis DP (2014). Macrophage reporter cell assay for screening immunopharmacological activity of cell wall-active antifungals. Antimicrob. Agents Chemother..

[51] Clay CD, Strait RT, Mahler A, Khodoun MV, Finkelman FD (2018). Anti-FcγRIIB mAb suppresses murine IgG-dependent anaphylaxis by Fc domain targeting of FcγRIII. J. Allergy Clin. Immunol..

[52] Cen X (2019). TLR1/2 specific small-molecule agonist suppresses leukemia cancer cell growth by stimulating cytotoxic T lymphocytes. Adv. Sci..

[53] Farhat K (2008). Heterodimerization of TLR2 with TLR1 or TLR6 expands the ligand spectrum but does not lead to differential signaling. J. Leukoc. Biol..

[54] Brandenburg K (2000). Physicochemical characteristics of triacyl lipid A partial structure OM-174 in relation to biological activity. Eur. J. Biochem..

[55] Schromm AB (2000). Biological activities of lipopolysaccharides are determined by the shape of their lipid A portion. Eur. J. Biochem..

[56] Li M, Wang H, Li W, Xu XG, Yu Y (2020). Macrophage activation on “phagocytic synapse” arrays: Spacing of nanoclustered ligands directs TLR1/2 signaling with an intrinsic limit. Sci. Adv..

[57] Noursadeghi M (2008). Quantitative imaging assay for NF-κB nuclear translocation in primary human macrophages. J. Immunol. Methods.

