# Supplementary material for: Spatial organization of FcγR and TLR2/1 on phagosome membranes differentially regulates their synergistic and inhibitory receptor crosstalk
Source: Sci Rep. 2021 Jun 28;11:13430. doi: 10.1038/s41598-021-92910-9 (PMC8238967; doi:10.1038/s41598-021-92910-9)
Supplement: Supplementary file 2 — Supplementary Information 1. [file 41598_2021_92910_MOESM2_ESM.docx]

Supporting Information for

**Spatial Organization of FcγRs and TLR2/1 on Phagosome Membranes Differentially Regulates Their Synergistic and Inhibitory Receptor Crosstalk**

Wenqian Li^a,b^, Miao Li^a^, Stephen M. Anthony^c^ and Yan Yu^a^

^a^Department of Chemistry, Indiana University Bloomington, Bloomington, IN 47405;

^b^Department of Molecular and Cellular Biochemistry, Indiana University Bloomington, Bloomington, IN 47405;

^c^Department of Bioenergy and Defense Technology, Sandia National Laboratories, Albuquerque, NM 87123.

**This PDF file includes:**

SI Figures 1 to 2

SI Video 1 Legend


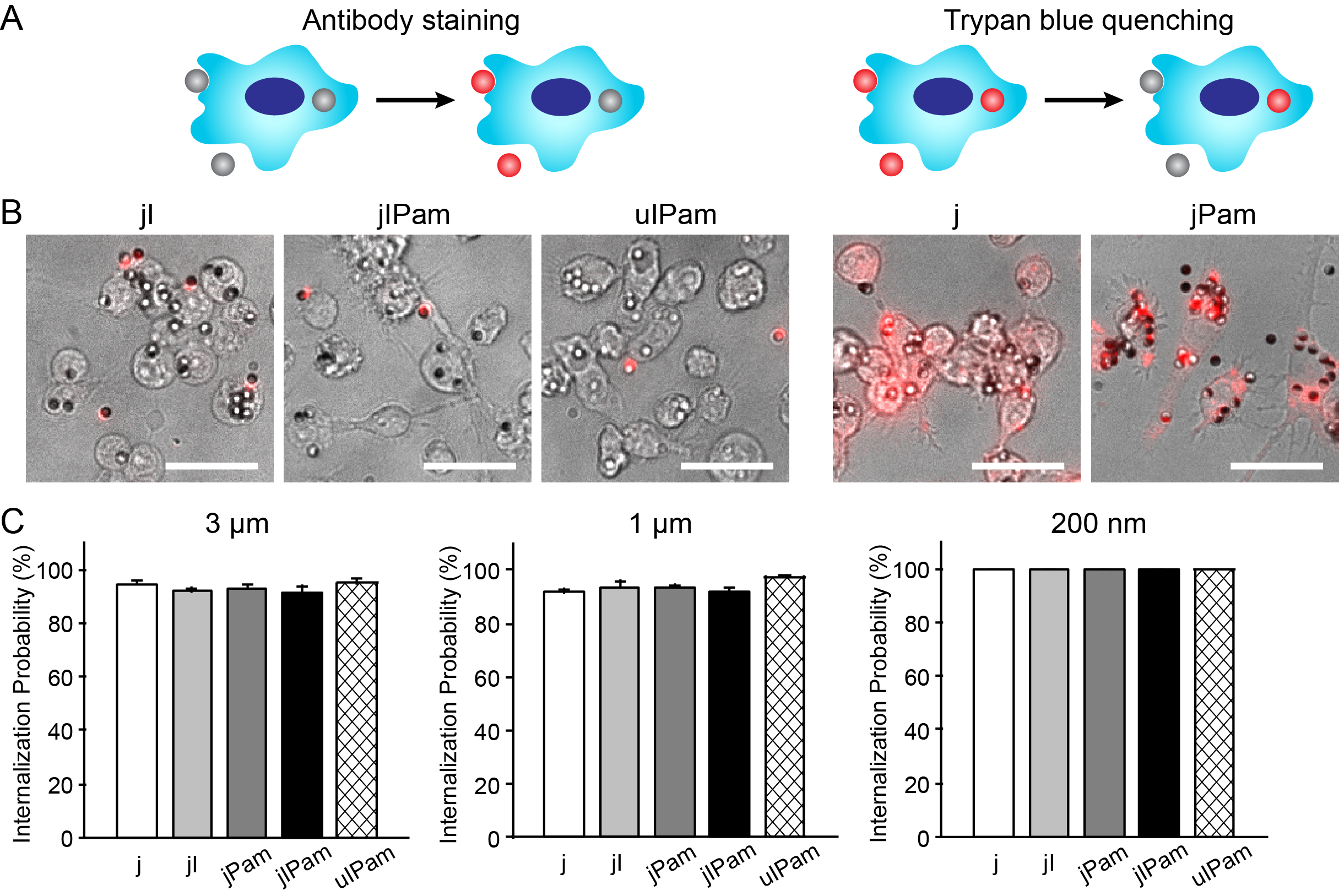


**SI Figure 1. Phagocytosis efficiency of different types of particles.** (A) Schematic illustration of two methods for measuring internalization probability of particles by macrophage cells: the antibody staining method for IgG-coated particles (left) and trypan blue quenching method for particles without IgG (right). In the antibody staining method, IgG on particles outside cells was labeled by fluorescently labeled secondary antibody (red). In the trypan blue quenching method, particles without IgG ligands were fluorescently labeled with Alexa 568 dyes. Fluorescence of particles outside cells was quenched by trypan blue, but those inside remained fluorescent (red). (B) Merged bright-field and fluorescence images showing the various types of particles (3 μm) and cells after antibody or trypan blue treatment. Scale bars: 30 μm. (C) Internalization probability of different types of particles by RAW 264.7 macrophage cells.


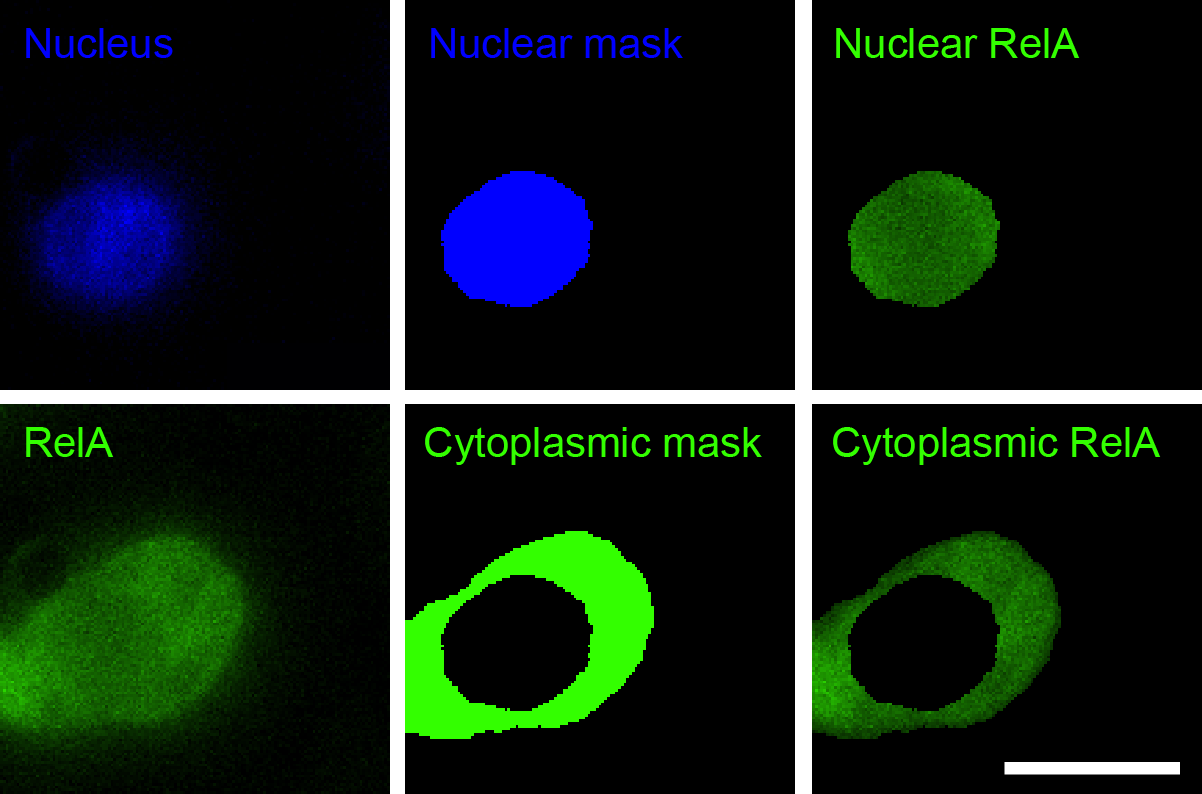


**SI Figure 2.** Snapshots showing image processing to calculate the nucleus-to-cytoplasm (Nuc/Cyt) fluorescence intensity ratio of RelA. Scale bar: 10 μm.

**SI Video**. A live-cell epi-fluorescence microscopy video showing RelA translocation in a RAW264.7 EGFP-RelA macrophage stimulated by a 3 μm Janus IgG/Pam3 (jIPam) particle. Pseudo colors in the video: RelA (green), nucleus (blue), IgG (red), and Pam3CSK4 (cyan).
